# Twelve-month surgical outcome and prognostic factors of stand-alone *ab interno* trabeculotomy in Japanese patients with open-angle glaucoma

**DOI:** 10.1371/journal.pone.0245015

**Published:** 2021-01-07

**Authors:** Takashi Omoto, Aya Sugiura, Takashi Fujishiro, Kimiko Asano-Shimizu, Koichiro Sugimoto, Rei Sakata, Hiroshi Murata, Ryo Asaoka, Megumi Honjo, Makoto Aihara

**Affiliations:** 1 Department of Ophthalmology, University of Tokyo Graduate School of Medicine, Bunkyo-ku, Tokyo, Japan; 2 Department of Ophthalmology, Tokyo Metropolitan Police Hospital, Nakano, Nakano-ku, Tokyo, Japan; Faculty of Medicine, Cairo University, EGYPT

## Abstract

The purpose of the study was to evaluate the 12-month surgical outcome and prognostic factors of stand-alone *ab interno* trabeculotomy. The changes in the intraocular pressure (IOP) and medication score and the success rate of the surgery were analyzed. Thirty-four eyes of 29 patients with primary open-angle glaucoma (POAG; n = 16) or pseudoexfoliation glaucoma (PEG; n = 18) with a 12-month follow-up period were included in the study. The decreases in IOP and medication score from the baseline to the all-time-point were statistically significant (*P* < 0.001). The surgical success rates were 97.1%, 76.5%, and 44.0% at 3 months (90 days), 6 months (180 days), and 12 months (365 days), respectively. A mixed effect Cox model revealed that the type of glaucoma (POAG) was significantly associated with surgical failure (*P* = 0.044). Furthermore, the surgical success rate was significantly higher in eyes with PEG than it was in those with POAG (*P* = 0.019). Stand-alone *ab interno* trabeculotomy significantly lowered both the IOP and the medication score in patients with glaucoma, although almost one quarter of the cases needed additional glaucoma surgeries. The surgical success rate was significantly higher in eyes with PEG than it was in those with POAG.

## Introduction

The demand for minimally invasive glaucoma surgery (MIGS), which can achieve a significant reduction in intraocular pressure (IOP) with fewer adverse events than do traditional surgical procedures, has been increasing [1–6]. Trabeculotomy is a type of glaucoma surgery that is used to cleave the trabecular meshwork (TM) and the inner wall of the Schlemm’s canal (SC), where the main sites of resistance to aqueous outflow are thought to reside [7–9]. In addition, the execution of this surgery via an *ab interno* approach using trabecular microhooks, such as the Kahook Dual Blade^®^ (New World Medical, CA, USA) [10], is considered a MIGS because it can be achieved through a single corneal incision without any conjunctival incision.

*Ab interno* trabeculotomy is often performed in combination with cataract surgery [11–19]; however, few studies have reported the clinical results of this approach, including series of stand-alone *ab interno* trabeculotomy surgery alone [20], with the exception of small case series of juvenile patients [21–23]. One possible explanation for this lack of studies is the achievement of sufficient reduction of IOP reduction or the number of medications needed is considered to be difficult in the absence of phacoemulsification. It is widely known that cataract surgery itself has a lowering effect on IOP and the number of medications needed in glaucoma patients [24–30]. However, a consensus has not been reached regarding the indications for this type of surgery. To address these issues, the aim of the current study was to evaluate the 12-month clinical results of stand-alone *ab interno* trabeculotomy at our institute, and to investigate the prognostic factors for surgical failure regarding IOP reduction.

## Methods

This observational study was approved by the institutional review board of the University of Tokyo and was conducted in accordance with the principles of the Declaration of Helsinki. Informed consent for the surgery was obtained from all patients.

### Subjects

Our inclusion criteria were *ab interno* trabeculotomy standalone surgery cases for POAG or PEG patients performed between January 2017 and December 2018 by 4 glaucoma specialists at the University of Tokyo Hospital and the patients who had a minimum of 12 months of routine follow-up. Our exclusion criteria were any other intraocular glaucoma surgery, such as *ab externo* trabeculotomy, in the past.

### Surgical technique

Our surgical technique was almost identical to that used in our previous study, with the exception of the exclusion of the cataract surgery [17]; in short, trabeculotomy was performed on the nasal side with a Kahook Dual Blade using the *ab interno* approach via a 2.4 mm temporal corneal incision. Blood reflux was confirmed in all cases intraoperatively and they were washed out with viscoelastic material at the end of the surgery. A topical antibiotic (moxifloxacin) and a corticosteroid (betamethasone sodium phosphate) were administered to the patient 4 times per day postoperatively; in addition, a miotic agent (pilocarpine hydrochloride) was provided to the patient 3 times per day. A miotic agents was also used for 5 times in 2 hours before the surgery. The doses of the drugs were tapered as the postoperative course advanced. On average, they were used for a month. Betamethasone was followed by fluorometholone for another month according to postoperative inflammation. IOP-lowering medications were stopped at the time of the surgery and were re-started based on the decision of the surgeon.

### Statistical analysis

A retrospective chart review was performed. Changes in IOP and medication scores over time were noted. IOP was measured by Goldmann applanation tonometry. The baseline IOP was measured as the mean of the final two measurements before the surgery. The use of combination therapy was scored as 2 and the oral administration of acetazolamide was scored as 1 in the medication score analysis. The postoperative routine use of the miotic agent was not counted as an IOP-lowering medication. These data were excluded from the analysis if re-operation had been performed. These values were compared between the baseline and each time point using the linear mixed model, in which the patients were regarded as a random effect. The linear mixed model adjusts for the hierarchical structure of the data, modeling it in a way in which measurements are grouped within subjects to reduce the possible bias of including both eyes of one patient [31, 32]. This was followed by Dunnett’s test for multiple comparisons [33].

The primary outcome of this study was the success rate of the surgery. Failure was defined as (1) an IOP > 21 mm Hg or a reduction <20% below the baseline on two consecutive follow-up visits after 3 months; or (2) reoperation for glaucoma, according to a previous report [34]. The surgical success rate was evaluated using the Kaplan–Meier method. We performed a multivariate Cox regression analysis to assess the potential association between various factors and surgical failure. This analysis was also performed using a mixed effect model (CRAN, coxme: Mixed Effects Cox Models, available at https://cran.r-project.org/web/packages/coxme/index.html, accessed in May 2020) [35]. The survival rates were compared using the log-rank test during the comparison of the surgical success rates between the two groups. Statistical significance was set at *P* < 0.05. All analyses were performed using the JMP^®^ Pro version 14 software (SAS Institute Inc., Cary, NC, USA) and the R software version 3.5.2. (The R Foundation for Statistical Computing, Vienna, Austria).

## Results

Thirty-four eyes of 29 patients with primary open angle glaucoma (POAG; n = 16) or pseudoexfoliation glaucoma (PEG; n = 18) were included in the study. The mean age at surgery was 69.6 ± 17.3 years. Specific patient information is provided in Table 1.

**Table 1:** Patient demographics.

Variables	Values
Eyes (patients)	34 (29)
Age, mean ± SD, years	69.6 ± 17.3
Types of glaucoma: POAG, PEG	16: 18
Lens status: phakia, pseudophakia	9: 25
Female:male ratio	21: 13
Right:left ratio	15: 19
Baseline	
IOP, mean ± SD, mmHg	25.6 ± 6.2
Medication score, mean ± SD	4.4 ± 1.1

POAG, primary open-angle glaucoma; PEG, pseudoexfoliation glaucoma; IOP, intraocular pressure

**Fig 1:**
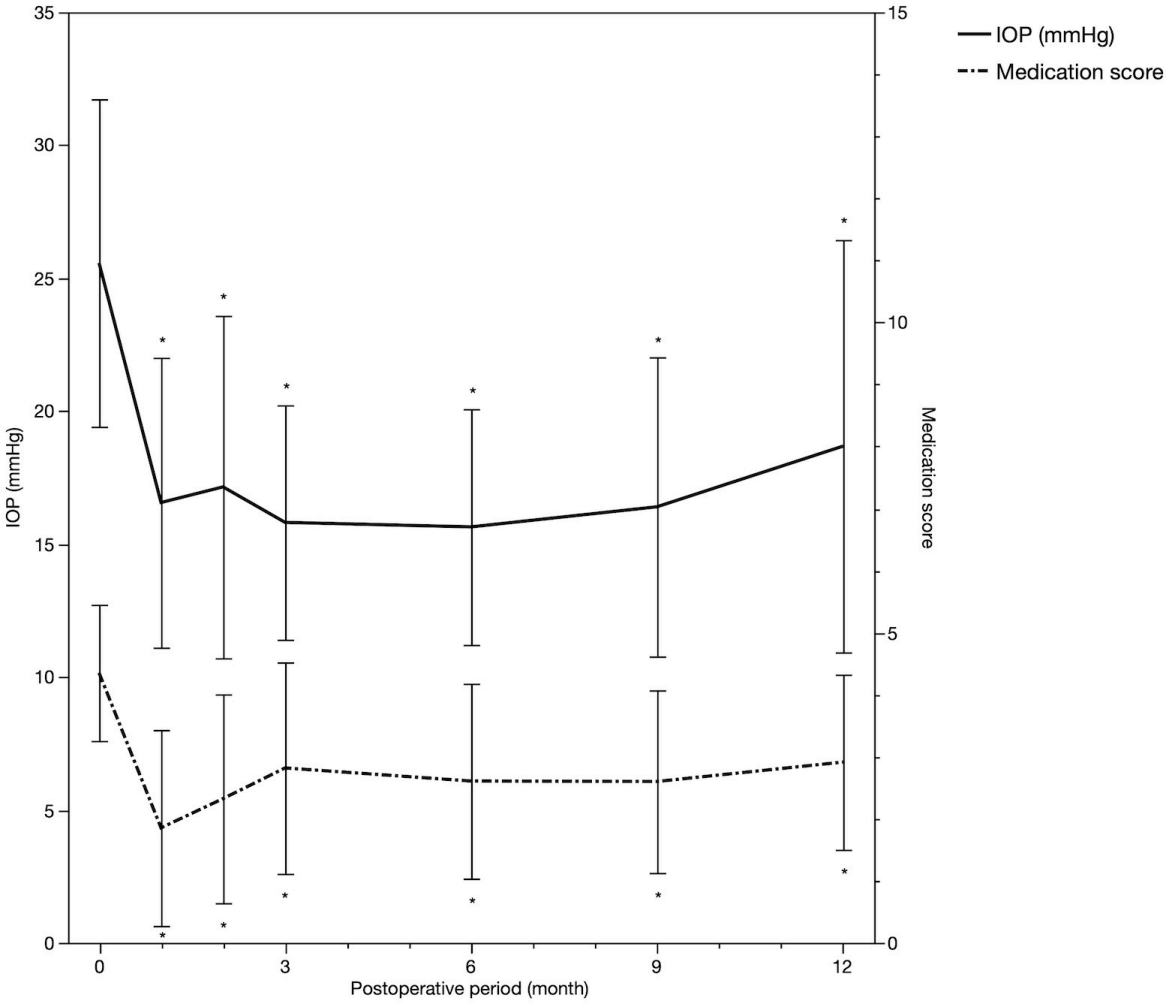
Changes in mean IOP and medication score over time. *Statistically significant difference between the baseline and each time point. Error bar, standard deviation. IOP, intraocular pressure.

The changes in IOP and medication score are shown in Fig 1. The decreases in IOP and medication score from the baseline (mean IOP, 25.6; medication score, 4.4) to the all-time-point (mean IOP, between 15.6 mmHg at 6 months and 18.8 mmHg at 12 months; mean medication score, between 1.9 at 1 month and 2.9 at 12 months) were statistically significant (*P* < 0.001, linear mixed model followed by Dunnett’s test). During this period, eight cases (23.5%) needed additional glaucoma surgeries; among them, seven cases underwent filtration surgeries, i.e., trabeculectomy with or without implantation of the Ex-PRESS^®^ glaucoma filtration device (Alcon Laboratories, Fort Worth, TX, USA), between 2 and 8 months, and one case underwent micro-pulse trans-scleral cyclophotocoagulation at 9 months after the initial surgery. Hyphema with niveau formation occurred in six eyes and a transient IOP spike developed in six eyes. No cases with hyphema needed wash out of them, spontaneously being absorbed within around 2 weeks. The visual acuity at postoperative 6 months (0.34 ± 0.57 LogMAR) was not statically different from the baseline value (0.34 ± 0.53 LogMAR, p = 0.92, paired t-test). In phakic eyes, there was one case with cataract progression followed by phacoemulsification at postoperative 5 months.

**Fig 2:**
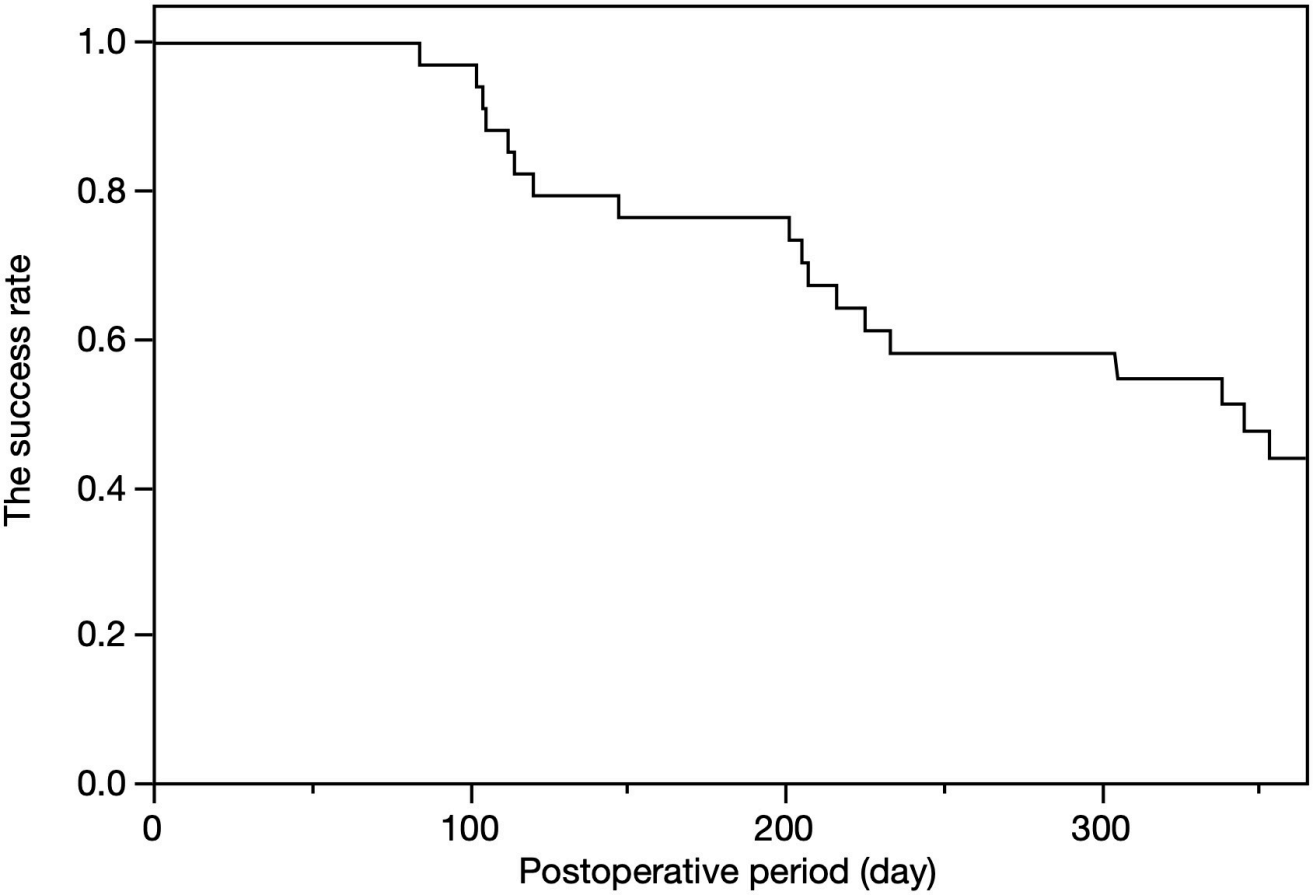
Success rate of the surgery, as assessed using the Kaplan–Meier method.

**Fig 3:**
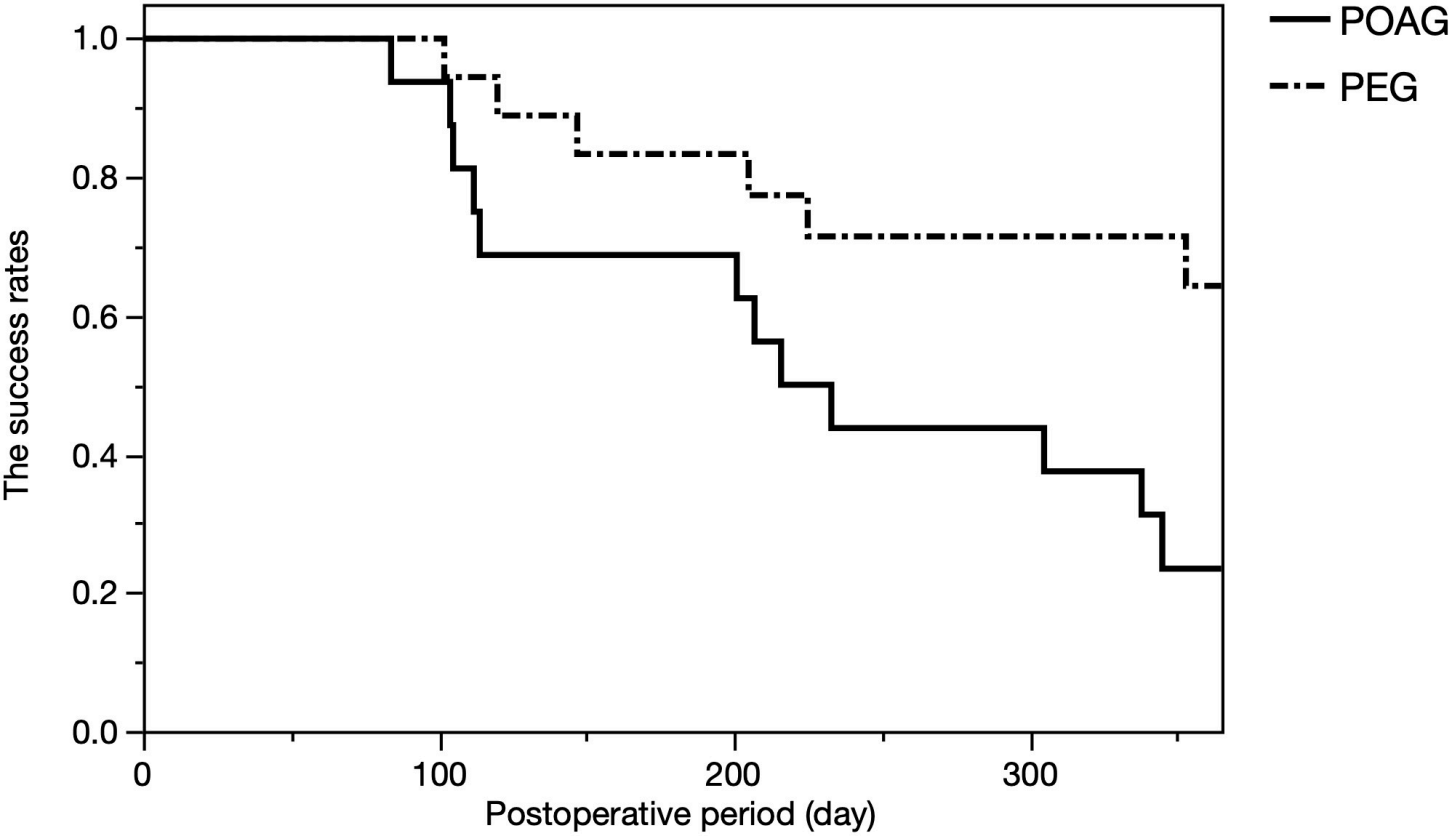
Comparison of the surgical success rates between patients with POAG and PEG. We observed a statistically significant difference between the two groups (*P* = 0.019, log-rank test). POAG, primary open-angle glaucoma; PEG, pseudoexfoliation glaucoma.

The results of the Kaplan–Meier survival analysis are shown in Fig 2. The surgical success rates were 97.1%, 76.5%, and 44.0% at 3 months (90 days), 6 months (180 days), and 12 months (365 days), respectively. The mixed effect Cox model revealed that the type of glaucoma (i.e., POAG) was significantly associated with surgical failure (*P* = 0.044, Table 2). Furthermore, the success rate was significantly higher in eyes with PEG than it was those with POAG (*P* = 0.019, log-rank test, Fig 3).

**Table 2:** The mixed effect Cox model.

	HR	95% CI	*P* value
Baseline IOP (per unit)	0.96	0.87–1.05	0.33
POAG/PEG	3.08	1.03–9.18	0.044*
Phakia/Pseudophakia	1.10	0.35–3.51	0.87

*P < 0.05

HR, hazard ratio; CI, confidence interval; IOP, intraocular pressure; POAG, primary open-angle glaucoma; PEG, pseudoexfoliation glaucoma

## Discussion

*Ab interno* trabeculotomy is an often-performed MIGS that uses trabecular microhooks, such as the Kahook Dual Blade used in the current study. This procedure is also called *ab interno* trabeculectomy because it enables not only the cleavage of the TM, but also the excision of the strip of TM. However, this surgery is often performed in combination with cataract surgery, with the exception of a study with a relatively short follow-up period (6 months) in mainly Caucasian patients (the IOP changed from 23.5 mmHg to 15.0 mmHg at 6 months) [20]. In a recent prospective study that compared two MIGS devices, i.e., the Hydrus Microstent (Ivantis, Inc, Irvine, CA) and the iStent Trabecular Micro-Bypass Stent System (Glaukos Corporation, San Clemente, CA), over a period of 12 months, the number of eyes achieving 20% reduction in IOP was 39.7% in the Hydrus group and 13.3% in the iStent group [36]. In that study, the IOP was reduced from 19.0 (27.5; after washout of the medications) to 17.3 mmHg, and from 19.1 (27.3; after washout of the medications) to 18.1 mmHg, respectively [36]. In the current study, we reported the clinical results of stand-alone *ab interno* trabeculotomy surgery with follow-up up to 1 year. The mean IOP and medication score were significantly decreased from the baseline, and these changes were maintained over the 1-year follow-up. The reduction in IOP observed in the current study (from 25.6 to 15.6 mmHg at 6 months and 18.7 mmHg at 12 months, with 14 out of 34 eyes achieving a 20% reduction at 12 months) was comparable to that of these past reports.

However, eight out of 34 cases (23.5%) needed additional glaucoma surgeries in the current study. This might be attributable to the severity of the disease in the patients included in the current study. In fact, the number of medications at the baseline in our study (4.4) was much higher than that of a past study (2.5), because those authors limited the number of medications to up to three drugs in their inclusion criteria. The eight eyes that required re-surgery here were four cases of POAG and four cases of PEG, with a baseline IOP of 27.4 mmHg and a baseline medication score of 4.6. Even though several of these severe cases resulted in additional surgeries, we could perform filtration surgeries with a non-damaged conjunctiva postoperatively.

At our institute, only 1 out of 32 cases of combined *ab interno* trabeculotomy resulted in additional surgery during the 1-year follow-up [37]. Therefore, it was very important to assess possible factors that are associated with surgical failure in the current study. As a result, PEG exhibited a better survival rate than did POAG in both multivariate (Table 2) and univariate (Fig 3) analyses. This is in an agreement with previous studies reporting favorable clinical results of other MIGS procedures in patients with PEG [38, 39]. PEG is a common cause of secondary glaucoma and it is reportedly frequent in many populations [40–44]. Moreover, PEG tends to be clinically more severe than is normal POAG [43, 45]. Interestingly, in the current study, the surgical outcomes were better in patients with PEG, which might be attributable to the mechanism of the disease; the deposition of extracellular material within the TM causes an aqueous outflow obstruction, resulting in an increased IOP [43, 46]. Hence, direct cleaving using a Kahook Dual Blade on site should be effective and PEG should be a good indication for this type of surgery.

In a recent study, a higher rate of re-operation was noted in eyes with a higher preoperative IOP after *ab interno* trabeculotomy surgery mostly combined with cataract surgery [19]. This is in contrast with our result of absence of significant changes in IOP after surgery (Table 2). This might be attributable to the small sample size used here, which was a limitation of this study. In addition, the functional parameters obtained from visual field tests and the morphological change such as cup-to-disc ratio or optical coherence tomography parameters were not evaluated in the current study. Furthermore, due to the retrospective nature of the study, we could not exclude bias completely between the groups. Further investigation using these parameters with larger case series and longer follow-up period is needed.

In conclusion, stand-alone *ab interno* trabeculotomy significantly lowered both IOP and medication score in patients with glaucoma, although almost one quarter of the cases needed additional glaucoma surgeries. The surgical success rate was significantly higher in eyes with PEG than it was in those with POAG.
